# The Tree-Drawing Test (Koch's Baum Test): A Useful Aid to Diagnose Cognitive Impairment

**DOI:** 10.1155/2015/534681

**Published:** 2015-06-15

**Authors:** Michelangelo Stanzani Maserati, Corrado Matacena, Luisa Sambati, Federico Oppi, Roberto Poda, Maddalena De Matteis, Roberto Gallassi

**Affiliations:** ^1^IRCCS Istituto delle Scienze Neurologiche di Bologna, 40139 Bologna, Italy; ^2^General Hospital of Imola, Cognitive Disorders Center, Imola, 40026 Bologna, Italy; ^3^Dipartimento di Scienze Biomediche e NeuroMotorie, Università di Bologna, 40123 Bologna, Italy

## Abstract

*Objective*. To study the Tree-Drawing Test in a group of demented patients and compare it with a group of mild cognitively impaired patients (MCI) and controls. *Methods*. Consecutive outpatients were classified as affected by dementia (Alzheimer's disease (AD), frontotemporal dementia (FTD), and vascular dementia (VD)) or by MCI. Patients and controls underwent the Tree-Drawing Test and MMSE. *Results*. 118 AD, 19 FTD, 46 VD, and 132 MCI patients and 90 controls were enrolled. AD patients draw trees globally smaller than other patients and controls. FTD patients draw trees with a wider space occupation than AD and MCI patients but smaller than controls as well as VD patients. Trees drawn by MCI patients are intermediate in size between AD patients and controls. The trunk-to-crown ratio of trees drawn by cognitive impaired patients is greater than controls while the tree size-relative-to-page space index is significantly smaller. The tree size-relative-to-page space index of trees drawn by AD patients is smaller than that of the other cognitively impaired patients. Tree height and the trunk-to-crown ratio are independent predictors of cognitive impairment. *Conclusions*. Trees drawn by cognitively impaired patients are different from those drawn by healthy subjects with a progressive differentiation from mild to more relevant degrees of cognitive impairment.

## 1. Introduction

The Tree-Drawing Test (TDT, Koch's Baum Test) is a projective psychological examination often used for assessing personality in the developmental age [1]. Its easiness of administration makes it a useful tool to express self-image and emotional states with relatively little resistance. TDT has been widely studied in schizophrenic patients showing a good capacity to distinguish pathological condition from normal condition [2–4]. Few studies have reported TDT in the elderly and in cognitively impaired patients suggesting it as a useful tool to assess mental functions in these groups [5–10]. To evaluate TDT differences between different types of cognitive deterioration, we studied TDT in a group of Alzheimer's disease (AD), frontotemporal dementia (FTD), and vascular dementia (VD) patients and compared it with a group of mild cognitive impairment (MCI) patients and controls.

## 2. Patients and Methods

We evaluated consecutive outpatients referred over a year's period by their relatives and physicians or who spontaneously presented themselves to the Cognitive Disorders Center of IRCCS Istituto delle Scienze Neurologiche of Bologna and to the Cognitive Disorders Center of the General Hospital of Imola, Italy. All subjects gave their informed consent to the study according to the Declaration of Helsinki. Patients were classified as demented or not according to DSM-IV-TR criteria [11]. Classification of dementia (AD, FTD, and VD) and diagnosis of MCI were based on the international criteria [12–15]. A group of controls, matched for age and education, was selected among relatives of patients.

**Figure 1:**
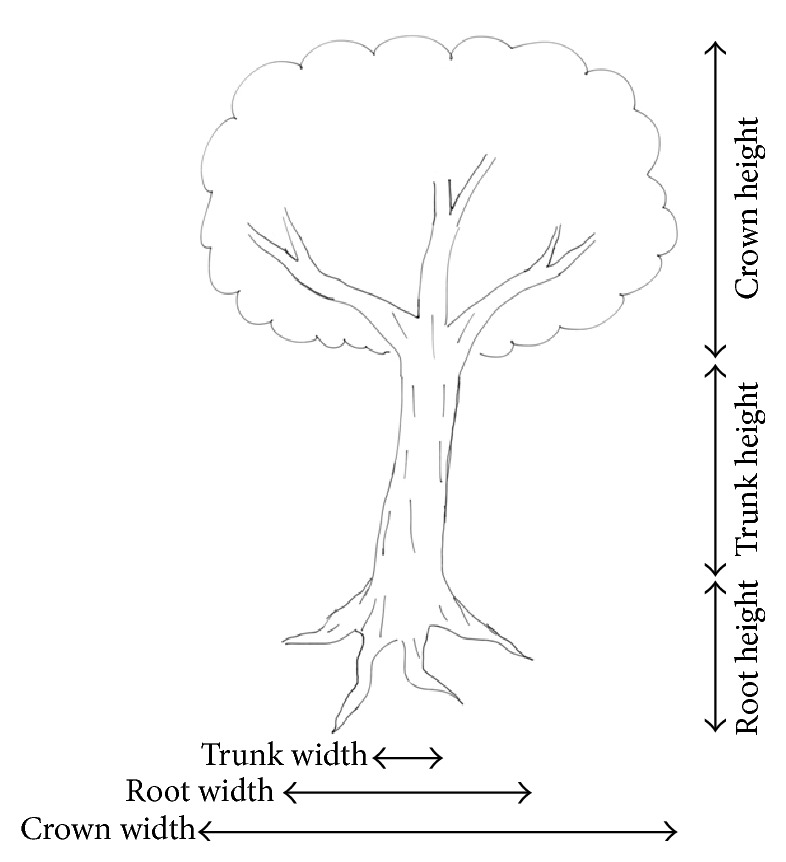
The Tree-Drawing Test: measurement of the height and width of crown, roots, and trunk.

All patients and controls were requested to draw a tree on an A4-sized white paper sheet with a pencil. Instructions were as follows: “Draw a tree, as you like.” No limits of time were given. The tree drawn was evaluated qualitatively (presence of crown, roots, branches, leaves, and flowers; types of trunk-end-opening, i.e., the top-end of the trunk, closed, opened, or wider than trunk) and quantitatively (height and width of trunk, crown, roots, and the trunk's tilt). The qualitative analysis of trunks, crowns, and branches included also the characterization of the shape (single or double lines for trunk and branches; open or closed crown). Heights and widths were obtained directly in millimeter units according to the criteria shown in Figure 1. Trunk's tilt was obtained in degrees by means of a goniometer. The trunk-to-crown ratio ((trunk height/crown height) × 10), the crown ratio (crown width/crown height), and the tree size-relative-to-page space index ((tree height × tree width)/(paper sheet height × paper sheet width)) were calculated. Mini Mental State Examination (MMSE) [16] was administered to patients and controls by an examiner blind to patient's diagnosis and TDT results. MMSE score was corrected for age and education according to Italian standardizations [17, 18].

Data were analyzed using the SPSS statistical analysis software, version 21.0. We performed a descriptive analysis of the various parameters of the patient groups; the comparisons of variables of various groups of the patients were obtained employing the multivariate general linear model with Bonferroni's correction with the significance level set at *P* = 0.05 and sex, age, education, and disease duration as covariates. In addition, multiple linear regression analyses with forward variable selection were used to investigate predictors of cognitive impairment.

## 3. Results

118 AD patients, 19 FTD, 46 VD, 132 MCI, and 90 controls were enrolled. Mean age, education, sex distribution, and disease duration of each group are listed in Table 1.

### 3.1. Qualitative Analysis

Qualitative characteristics of trees drawn by patients and controls are listed in Table 2. No significant differences emerge among groups.

### 3.2. Quantitative Analysis

**Figure 2:**
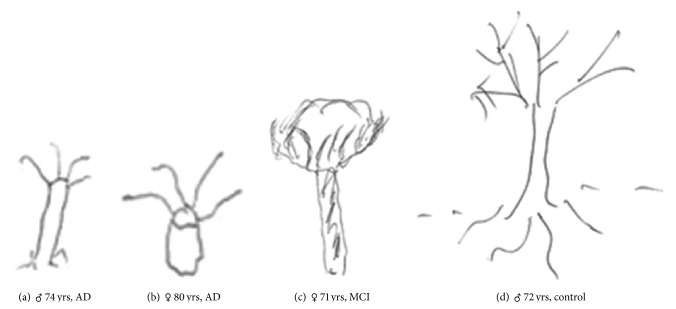
Examples of trees drawn by AD ((a) and (b)) and MCI (c) patients and controls (d).

Overall considering heights and widths of trunks, crowns, and roots of trees drawn by patients, significant differences emerge (Table 3). AD patients draw trees different from controls with respect to all variables, except crown ratio and tilt; trees drawn by AD patients are also globally smaller with respect to the other patients, demented or with MCI (Figure 2). FTD patients draw trees with a wider space occupation than AD and MCI but smaller than controls as well as VD patients because of reduced crown dimensions. Trees drawn by MCI patients are globally smaller than those drawn by controls and intermediate in size compared to those drawn by AD patients and healthy subjects.

The trunk-to-crown ratio of trees drawn by demented and MCI patients is greater than controls while the tree size-relative-to-page space index is significantly smaller. Furthermore, the tree size-relative-to-page space index of trees drawn by AD patients is smaller than that of the other cognitively impaired patients, demented or with MCI. The same index of trees drawn by FTD patients differs from AD and MCI but not from VD patients. The crown ratio of trees drawn by VD patients is significantly greater than controls.

Multiple linear regression model with forward variable selection includes, as independent predictors of cognitive impairment, the variables of tree height and trunk-to-crown ratio.

## 4. Discussion

TDT has been analyzed in the elderly and in cognitively impaired patients [5–10]. Authors overall find that elderly, and mostly cognitively impaired patients, draw small size trees of bad forms [7, 8] with a drawing space progressively smaller from normal to demented level [6]. In general, they do not distinguish different types of dementia, except for the Alzheimer type [5], and suggest TDT as a useful tool to evaluate mental functions of the elderly [7–10].

In our sample, cognitively impaired patients draw trees smaller with respect to healthy subjects. Trees drawn by AD patients in particular are significantly smaller with respect to trees drawn by other cognitively impaired patients, demented or MCI, and by controls. With respect to controls, AD patients draw smaller poorly detailed trees with an increased trunk-to-crown ratio and a reduced tree size-relative-to-page space index, that is, with a smaller crown and with a reduced space occupation. MCI patients draw trees intermediate in size between AD patients and healthy subjects suggesting a sort of progression from mild to greater degrees of cognitive impairment. Different tree dimensions with respect to controls in AD and MCI patients are related both to an increasing of the trunk-to-crown ratio and to a reduction of the tree size-relative-to-page space index, which is to a prevalence of the trunk with respect to the crown of the tree and to a global reduced space occupation. FTD patients differ from AD and MCI for the tree size-relative-to-page space index: they draw, in fact, trees bigger than AD and MCI patients but smaller than controls. Conversely, this significant difference does not exist between FTD and VD patients.

Globally, the trunk-to-crown ratio and the tree size-relative-to-page space index distinguish cognitively impaired patients, demented or not, from controls and the tree size-relative-to-page space index distinguishes also FTD patients from AD and MCI patients. Furthermore, the total tree height and the trunk-to-crown ratio are predictors of cognitive impairment in our patient sample.

The trunk-to-crown ratio is known to be inversely correlated to the development of linguistic abilities and abstract thinking during the course of the development [1, 19, 20]. Similarly, our patients show a trunk-to-crown ratio significantly greater with respect to controls with an increase from MCI patients to demented patients suggesting that linguistic abilities and abstract thinking gradually deteriorate with the worsening of cognitive functions. Furthermore, the reduction of the total tree size in demented and MCI patients could also be explained by the progressive impairment of constructional praxis and visuospatial functions along the course of cognitive deterioration. Finally, considering the progressive tree size reduction in cognitively impaired patients as an expression of a lower self-consciousness [1], a sort of personality regression could be speculated.

In adults, TDT had been also studied in psychiatric patients (eating disorders and schizophrenia) [2–4, 21]. Patients with eating disorders draw mostly smaller trees than controls, both in the total size and in the width of the trunk [21]. In schizophrenics, tree is small too and the trunk and the branches are mostly of single line type [3, 4].

So both psychiatric and cognitively impaired patients equally tend to draw smaller and bad formed trees than healthy individuals. However, some characteristics are different between the two groups such as the top-end of the trunk which is generally closed in healthy individuals and in cognitively impaired patients while it is typically opened in schizophrenics [3, 22, 23], possibly indicating a confusion between self and nonself [1].

Our data should be further confirmed in a wider patient sample selected according to advanced criteria of Alzheimer's disease and supported by biomarkers results and advanced neuroimaging techniques.

In conclusion, we think that TDT could be a useful tool for orienting cognitive impairment diagnosis and it could be an easy test to be administered by general practitioners and in specialized outpatient clinics. Furthermore, it could be included in extensive neuropsychological batteries exploring cognitive functions of cognitively impaired patients to attempt a possible and simple approach to the study of normal and pathological aging.

## Figures and Tables

**Table 1:** Clinical data of patients and controls.

	AD	FTD	VD	MCI	Controls
	*n* = 118	*n* = 19	*n* = 46	*n* = 132	*n* = 90
Age (years)	76.73 ± 5.12	73.16 ± 7.87	77.2 ± 4.86	76.12 ± 5.65	75.11 ± 8.71
Sex (male/female)	34/84	10/9	24/22	60/72	32/58
Education (years)	5.61 ± 3.1	6 ± 2.66	5.85 ± 3.04	5.6 ± 3.15	6.69 ± 3.18
Dominance (right/left)	116/2	19/0	46/0	125/7	85/5
Disease duration (years)	2.17 ± 0.99	1.84 ± 0.83	2.17 ± 0.85	1.36 ± 0.54	—

**Table 2:** Qualitative characteristics of trees.

	AD	FTD	VD	MCI	Controls
	*n* (%)	*n* (%)	*n* (%)	*n* (%)	*n* (%)
Trunk shape (s: single line, d: double line)	s: 23 (19%) d: 95 (81%)	s: 2 (11%) d: 17 (89%)	s: 8 (17%) d: 38 (83%)	s: 11 (9%) d: 121 (91%)	s: 5 (6%) d: 85 (94%)

Trunk end opening (0: top of the trunk closed, 1: top of the trunk opened, 2: top of the trunk wider than trunk)	0: 102 (86%) 1: 12 (10%) 2: 4 (4%)	0: 19 (100%) 1: 0 (0%) 2: 0 (0%)	0: 43 (93%) 1: 2 (4%) 2: 1 (3%)	0: 124 (94%) 1: 8 (6%) 2: 0 (0%)	0: 82 (91%) 1: 7 (8%) 2: 1 (1%)

Crown shape (o: open, c: closed, n: not done)	o: 86 (73%) c: 29 (25%) n: 3 (2%)	o: 12 (63%) c: 7 (37%)	o: 33 (72%) c: 13 (28%)	o: 106 (80%) c: 26 (20%)	o: 53 (59%) c: 37 (41%)

Branches shape (s: single line, d: double line, n: not done)	s: 84 (71%) d: 3 (3%) n: 31 (26%)	s: 11 (58%) d: 1 (5%) n: 5 (37%)	s: 30 (65%) d: 5 (11%) n: 11 (24%)	s: 101 (77%) d: 5 (4%) n: 26 (19%)	s: 53 (59%) d: 13 (14%) n: 24 (27%)

Leaves	24 (20%)	5 (26%)	9 (20%)	41 (31%)	28 (31%)

Flowers	0	0	2 (4%)	6 (5%)	4 (4%)

**Table 3:** Quantitative analysis of trees and significant differences between groups.

	AD	FTD	VD	MCI	Controls	Controls versus				AD versus			FTD versus		VD versus
	Mean ± SD	Mean ± SD	Mean ± SD	Mean ± SD	Mean ± SD	AD	FTD	VD	MCI	FTD	VD	MCI	VD	MCI	MCI
MMSEc	17.61 ± 3.89	18.68 ± 6.42	19.52 ± 3.28	26.09 ± 1.77	28.04 ± 1.42	∗∗∗∗	∗∗∗∗	∗∗∗∗	∗∗∗∗	n.s.	∗∗∗	∗∗∗∗	n.s.	∗∗∗∗	∗∗∗∗
Trunk-to-crown ratio	12.62 ± 7.59	12.75 ± 4.91	12.29 ± 6.12	11.04 ± 5.37	8.35 ± 3.72	∗∗∗∗	∗∗	∗∗∗	∗∗∗	n.s.	n.s.	n.s.	n.s.	n.s.	n.s.
Crown ratio	1.51 ± 0.61	1.63 ± 0.57	1.68 ± 0.73	1.53 ± 0.45	1.45 ± 0.48	n.s.	n.s.	∗	n.s.	n.s.	n.s.	n.s.	n.s.	n.s.	n.s.
Tree size-relative-to-page space	0.03 ± 0.03	0.13 ± 0.11	0.1 ± 0.68	0.08 ± 0.06	0.23 ± 0.19	∗∗∗∗	∗∗∗∗	∗∗∗∗	∗∗∗∗	∗∗∗∗	∗∗∗∗	∗∗∗∗	n.s.	∗∗∗∗	∗
Tree height	52.09 ± 24.04	109.89 ± 38.98	91.41 ± 28.35	80.77 ± 28.87	132.61 ± 57.81	∗∗∗∗	∗∗	∗∗∗∗	∗∗∗∗	∗∗∗∗	∗∗∗∗	∗∗∗∗	∗	∗∗∗∗	∗∗
Tree width	35.86 ± 21.14	70 ± 39.08	65.43 ± 26.86	56.67 ± 25.6	96.57 ± 41.67	∗∗∗∗	∗∗∗∗	∗∗∗∗	∗∗∗∗	∗∗∗∗	∗∗∗∗	∗∗∗∗	n.s.	∗	∗
Trunk height	26.64 ± 15.34	56.74 ± 22.83	45.87 ± 21.11	38.11 ± 17.71	52.73 ± 23.74	∗∗∗∗	n.s.	n.s.	∗∗∗∗	∗∗∗∗	∗∗∗∗	∗∗∗∗	∗	∗∗∗∗	∗
Trunk width	8.13 ± 7.25	13.58 ± 10.32	11.02 ± 7.99	9.85 ± 6.84	13.23 ± 7.78	∗∗∗∗	n.s.	n.s.	∗∗	∗∗	∗	n.s.	n.s.	n.s.	n.s.
Trunk's tilt	89.39 ± 8.79	86.89 ± 4.65	88.09 ± 5.64	89.59 ± 6.14	90.7 ± 4.21	n.s.	n.s.	n.s.	n.s.	n.s.	n.s.	n.s.	n.s.	n.s.	n.s.
Crown height	23.86 ± 13.11	47.47 ± 21.12	41.04 ± 15.67	38.34 ± 16.47	72.46 ± 39.22	∗∗∗∗	∗∗∗∗	∗∗∗∗	∗∗∗∗	∗∗∗∗	∗∗∗∗	∗∗∗∗	n.s.	∗	n.s.
Crown width	34.71 ± 21.86	75.11 ± 39.53	64.8 ± 26.58	56.48 ± 25.54	95.91 ± 41.22	∗∗∗∗	∗∗	∗∗∗∗	∗∗∗∗	∗∗∗∗	∗∗∗∗	∗∗∗∗	n.s.	∗∗	n.s.
Root height	1.59 ± 4.63	5.68 ± 8.7	4.5 ± 8.45	4.31 ± 7.83	7.42 ± 13.34	∗∗∗∗	n.s.	∗	∗∗	∗	∗	∗	n.s.	n.s.	n.s.
Root width	3.53 ± 12.13	15.84 ± 23.13	9.41 ± 16.48	9.33 ± 16.89	18.44 ± 32.19	∗∗∗∗	n.s.	∗∗	∗∗∗∗	∗∗	n.s.	∗∗	n.s.	n.s.	n.s.

^****^ *P* < 0.000; ^***^ *P* < 0.001; ^**^ *P* < 0.01; ^*^ *P* < 0.05; n.s.: not significative.
